# Identifying Modules of Coexpressed Transcript Units and Their Organization of *Saccharopolyspora erythraea* from Time Series Gene Expression Profiles

**DOI:** 10.1371/journal.pone.0012126

**Published:** 2010-08-12

**Authors:** Xiao Chang, Shuai Liu, Yong-Tao Yu, Yi-Xue Li, Yuan-Yuan Li

**Affiliations:** 1 Key Lab of Systems Biology, Bioinformatics Center, Shanghai Institutes for Biological Sciences, Chinese Academy of Sciences, Shanghai, China; 2 Shanghai Center for Bioinformation Technology, Shanghai, China; 3 Graduate School of the Chinese Academy of Sciences, Beijing, China; 4 Test Center for Agriculture Quality of Jinan, Jinan, Shandong, China; University of Nottingham, United Kingdom

## Abstract

**Background:**

The *Saccharopolyspora erythraea* genome sequence was released in 2007. In order to look at the gene regulations at whole transcriptome level, an expression microarray was specifically designed on the *S. erythraea* strain NRRL 2338 genome sequence. Based on these data, we set out to investigate the potential transcriptional regulatory networks and their organization.

**Methodology/Principal Findings:**

In view of the hierarchical structure of bacterial transcriptional regulation, we constructed a hierarchical coexpression network at whole transcriptome level. A total of 27 modules were identified from 1255 differentially expressed transcript units (TUs) across time course, which were further classified in to four groups. Functional enrichment analysis indicated the biological significance of our hierarchical network. It was indicated that primary metabolism is activated in the first rapid growth phase (phase A), and secondary metabolism is induced when the growth is slowed down (phase B). Among the 27 modules, two are highly correlated to erythromycin production. One contains all genes in the erythromycin-biosynthetic (ery) gene cluster and the other seems to be associated with erythromycin production by sharing common intermediate metabolites. Non-concomitant correlation between production and expression regulation was observed. Especially, by calculating the partial correlation coefficients and building the network based on Gaussian graphical model, intrinsic associations between modules were found, and the association between those two erythromycin production-correlated modules was included as expected.

**Conclusions:**

This work created a hierarchical model clustering transcriptome data into coordinated modules, and modules into groups across the time course, giving insight into the concerted transcriptional regulations especially the regulation corresponding to erythromycin production of *S. erythraea*. This strategy may be extendable to studies on other prokaryotic microorganisms.

## Introduction

*Saccharopolyspora erythraea*, formerly known as *Streptomyces erythraeus*, is a gram-positive, spore-forming bacterium. It is used for industrial-scale production of erythromycin A, a broad-spectrum antibiotic against Gram-positive bacteria [1]. Due to the commercial importance of erythromycin and its derivatives, intensive efforts have been devoted to its biosynthesis mechanism, aiming to increase strain productivity [2], [3]. The complete genome sequences of *S. erythraea* strain NRRL2338 was released in 2007 [1], and indicated considerable divergence of *S. erythraea* from the streptomycetes in gene organization and function.

In prokaryotic genomes, a set of genes and their associated regulatory elements are grouped into an operon, and co-transcribed as a single unit, or transcript unit (TU); a group of genes and operons subject to the regulation by the same transcription factors are defined as a regulon; at a much higher level, the genes, operons and regulons controlled by a set of transcriptional factors (TFs) at certain times under certain conditions within the cell are termed as a stimulon or modulon [4], [5]. The hierarchical transcriptional regulatory networks have been shown to meet the inherent property of cell regulation mechanisms [6]. Analysis of microarray transcriptome data allows for identification of TU sets that share a similar expression profile across multiple temporal, environmental and genetic conditions, and these TUs are candidates for regulons and stimulons [7]. Transcriptome data are expected to give fresh impetus to the study of the hierarchical regulatory organization of microorganisms.

Recently, Peano and his colleagues designed a time series microarray experiment at ten different time points according to erythromycin production and cell growth curve [8]. The DNA microarray was constructed on the *S. erythraea* strain NRRL2338 genome sequence at whole transcriptome level, and the expression profiles of 6494 ORFs were monitored. The authors identified 404 most differentially expressed genes during time course characterizing three distinct phases: a rapid growth until 32 h (Phase A); a growth slowdown until 52 h (Phase B); and another rapid growth phase from 56 h to 72 h (Phase C). The erythromycin-biosynthetic (ery) gene cluster was confirmed to be up-regulated during Phase A [8]. These findings extend our understanding of how *S. erythraea* genes are transcriptionally regulated at a global level.

In the present work, we re-analyzed the transcriptome data from the angle of the hierarchical modular structure of bacterial transcriptional regulation. We constructed a multi-layer coexpression network by organizing genes into TUs, modules and groups step by step based on expression correlation. A total of 27 modules were identified from 1255 differentially expressed TUs across time course, which were further merged into 4 groups showing some specificity to cell growth phases. Functional enrichment analysis indicated the biological significance of our hierarchical network. Among the 27 modules, two are highly correlated to erythromycin production with non-concomitant checking showing more significant correlation. These two modules were found to have direct association by a following calculation aiming to reveal the hidden associations after removing the effects of the other modules. The landscape of the hierarchical coexpression network of *S. erythraea* shows the power of this approach investigating the regulatory mechanisms of *S.erythraea* and could be extended to the study of other microorganisms.

## Results

### Identification of operon structures and differentially expressed transcript units

**Figure 1:**
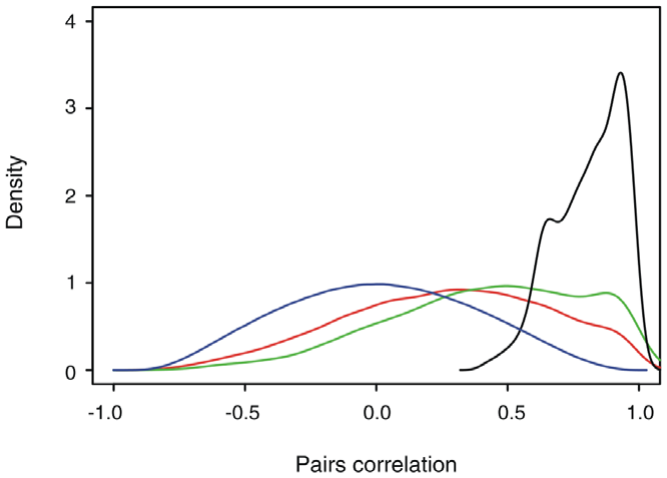
Density curves of pairs correlation. Density curves of the correlations of all pairs (blue), all adjacent pairs (red), all pairs within predicted operon pairs (green) and gene pairs within filtered operons (black).

In a typical bacterial genome, about half of genes are located in operons [9]. Since the operon structure affects the regulation of gene expression, operon prediction becomes the first step towards regulatory network reconstruction at the whole genome level. The predicted operons of *S. erythraea* strain NRRL2338 were obtained from DOOR [10]. Although the prediction program used by DOOR was ranked as the most accurate among 14 currently popular operon prediction programs [11], [12], it still generated false operon structures. We therefore screened the operons according to the expression correlation of within-operon gene pairs. When the average expression correlation of all possible within-operon pairs is below 0.6, or the correlation of any adjacent within-operon pair is below 0.4, this operon was filtered out. The expression correlations of all possible gene pairs, all adjacent gene pairs, all possible gene pairs within predicted operons and all possible gene pairs within filtered predicted operons were respectively plotted as density curves in Figure 1. With the curve of all pairs following an approximately normal distribution, the overall correlation of all pairs, all adjacent pairs and all pairs within predicted operon pairs is gradually increasing (Figure 1); and gene pairs within filtered operons present a distinctly higher correlation than the other three groups of gene pairs (Figure 1).

According to the common definition, both operons and genes not assigned to any operons, were regarded as transcript units, and the average expression value of within-operon genes was adopted to characterize the expression profile of the operon. We first identified the top 2000 differentially expressed TUs using EDGE and Timecourse separately, and the overlapped 1255 TUs, corresponding to 1668 genes (approximate 25% of 6494 ORFs), were identified as differentially expressed ones for further analysis. The overlapped differentially expressed TUs accounted for 60% of those generated by EDGE or Timecourse at almost every time points (Figure S1).

### Hierarchical clustering of transcript units into coexpression modules and groups

**Figure 2:**
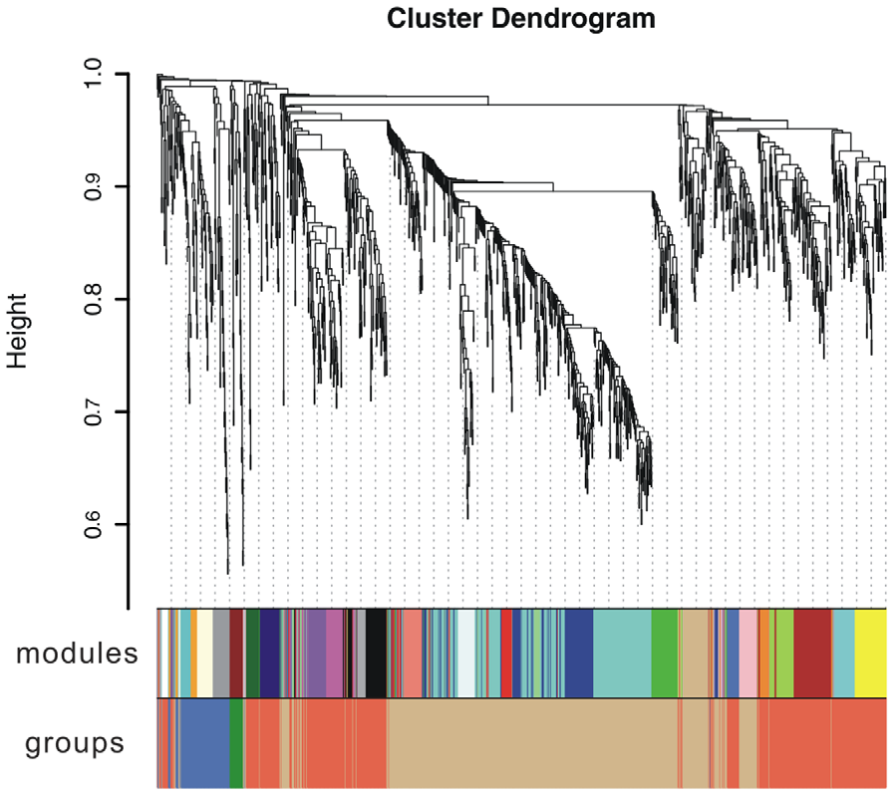
Hierarchical clustering results of the differentially expressed TUs. The upper section is the cluster dendrogram of TUs; the middle and lower section indicate the modules of coexpressed TUs and groups of coexpressed modules respectively.

**Figure 3:**
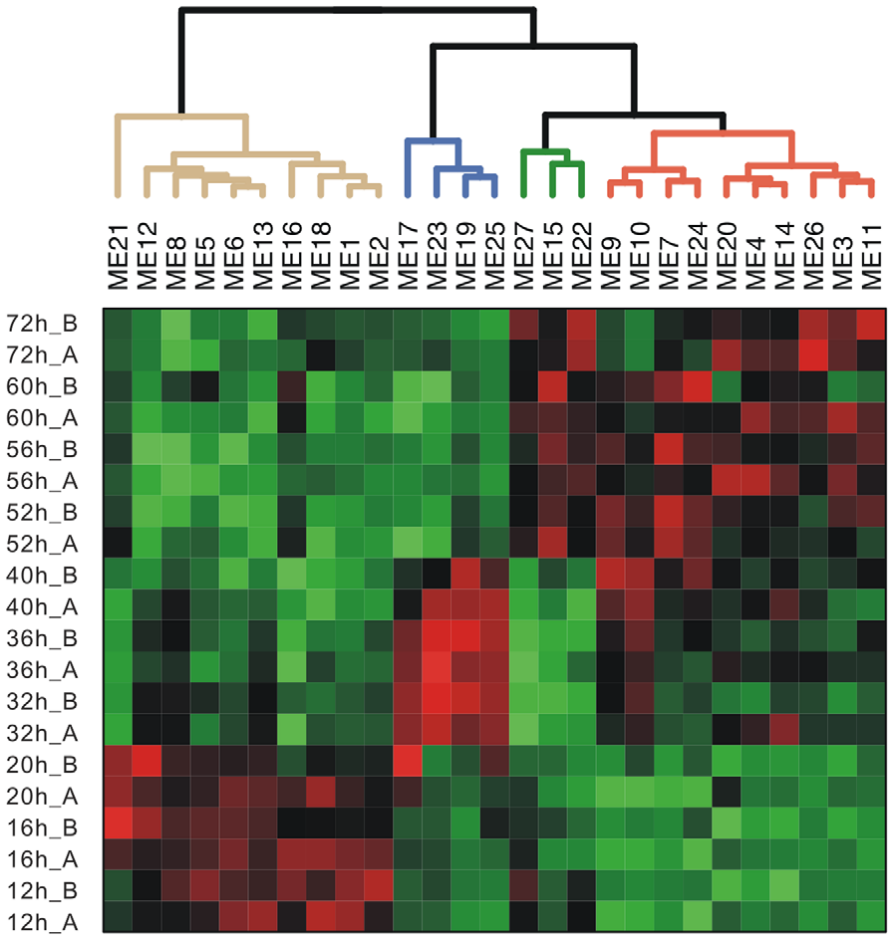
Global expression profiling of modules of coexpressed TUs during the growth time course. Each column of the heatmap corresponds to one module and each row corresponds to a sample. In the heatmap, green color represents down-regulation, while red represents up-regulation. The dendrogram obtained by hierarchical clustering are shown along the top. Branches of the dendrogram are colored in tan, blue, green, red, which correspond to group 1, 2, 3, 4 respectively.

After constructing weighted coexpression network based on differentially expressed TUs, the 1255 TUs were clustered into a total of 27 coexpression modules (Figure 2) ranging from 10 to 238 TUs in size. For each module, the eigengene was calculated to represent its expression profile along the time course, and the 27 modules were further clustered by computing the correlation between eigengenes (see methods for details). The height cut was set as 0.6 so that the resulting four groups of coexpressed modules accorded with the growth phases of *S. erythraea* [8] (Figure S3). In summary, group 1 are up-regulated in growth phase A (rapid growth); group 2 are dominantly up-regulated in phase B (growth slowdown); groups 3 and 4 together correspond to phase C (another rapid growth), with group 3 exclusively up-regulated in phase C, and group 4 up-regulated in both phase B and C (Figure 3).

The largest group, group 1, contains 672 TUs, involving 10 modules including two largest modules, module 1 and module 2. Group 4 is the second largest module, consisting of 416 TUs. Group 2 and group 3 contain 99 and 67 TUs respectively. It is noticeable that quite a few of TUs belonging to the same gene cluster for the biosynthesis of second metabolites were grouped into the same module. For example, all TUs in ery cluster for erythromycin biosynthesis were grouped to module 12, group 1; tpc1 cluster, nrps1 cluster and nrps3 cluster were included by module 19, group 2.

### Functional enrichment analysis of coexpression modules and groups

**Figure 4:**
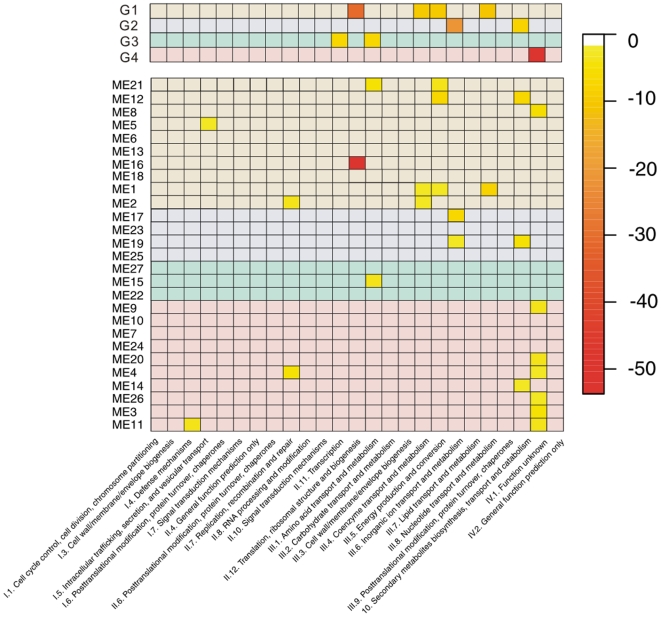
Functional enrichment analysis of modules and groups. The functional categories of the genes of each module were tested for enrichment by a hypergeometric test. The y-axis is the number of identified modules (upper matrix) and groups (lower matrix), the x-axis is the classification of COGs. The background of each cell is according to the groups (group 1 in tan, group 2 in blue, group 3 in green, group 4 in red), The p-value of each cell less than 0.01 is marked in colors according to the color legend.

Functional enrichment analysis based on COG category annotation provided by Oliynyk *et al.* [1] showed that two-thirds of the 27 modules are associated with one or more functional categories (Figure 4). For instance, module 1, the largest one, is assigned to III.4 Coenzyme transport and metabolism, III.5 Energy production and conversion, III.8 Nucleotide transport and metabolism. Module 12 which contains the ery gene cluster is related to III.5 Energy production and conversion, III.10 Secondary metabolites biosynthesis, transport and catabolism, while module 16 displays a strong association with II.12 Translation, ribosomal structure and biogenesis. Besides, several modules are found to be enriched in IV.1 Function unknown as a large number of genes in *S. erythraea* are designated as denovo.

The association relationship between coexpression modules and COG categories became much clearer after clustering modules into groups (Figure 4). The largest group, group 1, are enriched in II.12 Translation, ribosomal structure and biogenesis, III.4 Coenzyme transport and metabolism, III.5 Energy production and conversion, III.8 Nucleotide transport and metabolism. Group 2 is exclusively involved in III.6 Inorganic ion transport and metabolism, III.10 Secondary metabolites biosynthesis, transport and catabolism, indicating that secondary metabolites production is activated dominantly in phase B. Genes in group 3 are enriched in II.11 Transcription, III.1 Amino acid transport and metabolism. The second largest group, group 4 is assigned to IV.1 Function unknown, suggesting unknown complicated mechanisms related to this group.

### Estimation of the correlation between erythromycin production and coexpression modules or TUs

To investigate the global regulation mechanism of erythromycin production, we examined the correlation between erythromycin productivity and expression levels of modules and TUs. We applied a fifth-order polynomial fit to the time series data of erythromycin production, and the first derivative of the fitted polynomial curves was calculated to approximately represent the erythromycin production rate. By computing the Pearson correlation coefficients between the eigengenes of 27 modules and erythromycin production rate respectively. we found that module 17 enriched with genes of III.6 Inorganic ion transport and metabolism category are most correlated to erythromycin production (cor = 0.911), whereas only a moderate correlation (cor = 0.672) were found between erythromycin production and module 12 which contains ery cluster. We assumed that there probably exists a delayed effect when genes are modulated to function in a certain process. Thus, we re-investigated the correlation by first shifting the production time series by an hour from 4 hours ahead to 6 hours behind and calculating the overlapping time point-to-time point correlations (Table S1). It was found that ery-containing module 12 became obviously correlated to erythromycin production when the time series of production lagged by 3 hours to that of gene expression, whereas module 17 partly lost its correlation to erythromycin production. In comparison, the correlation between module 17 and antibiotic production was strengthened when the time series was advanced.

We also calculated the correlation between expression profiles of TUs and erythromycin production rate respectively. Several glycolysis genes were found to be highly linked to erythromycin output including the maltose operon and its regulator (MalR).

### Organizational structure of coexpression modules

To reveal hidden direct correlations between modules, i.e correlations masked by the effect of other modules, we adopted partial correlation analysis and constructed a network based on Gaussian graphical model where nodes correspond to modules and edges indicate significant partial correlations between modules.

Interestingly, module 21 seems to play a hub role in group 1 as it connects to the two largest modules, module 1, module 2, as well as module 12 which contains the ery cluster. Additionally, module 12 also connects to module 17 in group 2, indicating an intrinsic association between the two erythromycin production correlated modules. The associations between groups 3 and 4, groups 1 and 2 imply the interaction of modules in adjacent phases.

## Discussion

In this study, we built a hierarchical coexpression network from time-course microarray gene expression data of *S. erythraea* by organizing coexpressed genes into TUs, modules and groups. Instead of clustering differentially expressed genes [8], we screened TUs according to within-TU expression correlation, and then identified 1255 differentially expressed TUs across time course, which are the basic elements of the following clustering procedures. The 1255 TUs correspond to 1668 genes, accounting for around 25% of 6494 ORFs. The proportion of 25% accords with the general criteria that top 25% genes are considered as significantly differentially expressed [13].

A total of 27 modules and 4 groups were identified from 1255 differentially expressed TUs across time course. Functional enrichment analysis indicated that almost all of these expression modules and groups have their dominant functions. To sum up, primary metabolism (dominant in group 1) is activated in phase A (rapid growth), and secondary metabolism (dominant in group 2) is induced in phase B (growth slowdown). When cells enter phase C, another rapid growth phase, transcription, amino acid transport and metabolism (dominant in group 3) are activated. It is interesting that group 4, up-regulated in both phase B and phase C (from 36h to 72 h), are enriched in the ‘function unknown’ category. We propose that this group may contain some *S. erythraea* specific genes which play particular roles in phases B and C, while have no orthologous gene in the public domain (non-redundant protein sequence database) and therefore were designated as ‘function unknown’.

The functional enrichment analysis on groups, as well as that on modules, implied the cooperation relationships among modules or groups and confirmed the biological relevance of our hierarchical network. This hierarchical coexpression network will provide insight into complicated global mechanisms of transcriptional regulations of *S. erythraea*, including regulation mechanism of erythromycin synthesis. The methodology of this work can be used in studies of other microorganisms.

We also tried several other clustering methods designed for time course expression data when we clustered differentially coexpressed TUs into modules [14]–[17]. For example, those developed by Madeira *et al.* (2009) and Kiddle *et al.* (2010). Meanwhile, comparisons with other recent methods was not possible due to different data basis [14] or unavailable resources [17]. It was found that the TU clustering results of different methods were basically consistent (Table S2).

**Figure 5:**
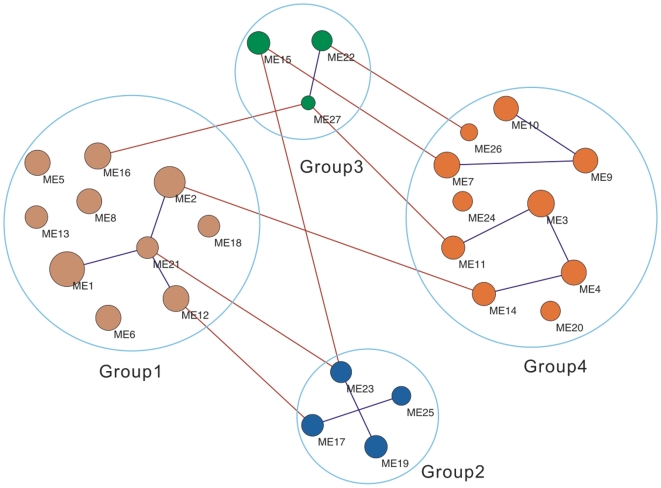
Network representation of modules of coexpressed TUs. modules of coexpressed TUs are represented by circles. The sizes of circles are corresponding to module sizes, and the colors of circles are corresponding to groups of coexpressed modules (group 1 in tan, group 2 in blue, group 3 in green, group 4 in red). Edges between modules in the same group are colored in blue, while edges between modules in different groups are colored in brown.

As the capability of producing erythromycin is the most significant commercial trait of *S. erythraea*, many efforts have been devoted to the gene regulation during erythromycin synthesis. Here, we made an attempt to clarify the factors related to erythromycin production by estimating the correlation between production and the expression profiles of coexpression modules. Two modules, module 12 and module 17, were found to be correlated to erythromycin production. Module 12 contains ery gene cluster while module 17 dominantly involves ABC transporter family. It is noticeable that by introducing time lag caused by the delayed effect of transcriptome, we found that the correlation between production and ery-containing module 12 achieved the highest value when the time series of production lagged by 3 hours. This observation is basically consistent with the common sense that the phenotype at the metabolome level lag behind the regulation at the transciptome level, that is, it is reasonable that erythromycin production profile lag behind the transcriptional regulation of related genes. While, the correlation between ABC transporter-containing module 17 and antibiotic production was increased only when the time series was advanced. Since ABC transporter family utilize the energy of ATP hydrolysis to transport various small molecules across cellular membranes, it seems that ABC transporters may mainly mediate the efflux of erythromycin rather than import molecules associated with erythromycin synthesis. The intrinsic association relationship between modules 12 and 17 was suggested by their correlation in the organizational structure of coexpression modules (Figure 5) inferred by estimating the partial correlation coefficients of the 27 modules.

In addition, we found some glycolysis genes highly correlated to erythromycin output, including genes related to maltose metabolism, such as maltose operon and its regulator (MalR), alpha-glucosidase hydrolyzing maltose to alpha-D-Glucose, and 6-phosphofructokinase catalyzing beta-D-Fructose 6-phosphate to alpha-D-Glucose 6-phosphate. Both alpha-D-Glucose and alpha-D-Glucose 6-phosphate could be transformed into alpha-D-Glucose 1-phosphate, which participates in the synthesis of erythromycin [18], suggesting that the assimilation and transformation of maltose may play an important role in erythromycin production. Noticeably, MalR may act as an activator of maltose in *S. erythraea* considering its high positive correlation with maltose operon expression, which is contradictory to the original annotation of MalR as a repressor [1].

We believe our correlation analyses provide clues for understanding the regulation mechanisms of erythromycin synthesis and bioengineering of related process aiming to increase the productivity. More significantly, the ‘time lag’ strategy could be utilized in integrating omics data when the delayed effect has to be considered.

## Materials and Methods

### Preparation of dataset

Time-course microarray data of *S. erythraea* strain NRRL2338 designed by Peano *et al* [8] were obtained from the GEO (Gene Expression Omnibus) repository (accession number: GSE9422) [19]. Predicted operon structures of *S. erythraea* strain NRRL2338 were downloaded from DOOR (Database for prOkaryotic OpeRons) [10], whose prediction program was recently ranked by an independent assessment as the most accurate among 14 operon prediction programs across all three performance measurements: sensitivity, specificity and overall accuracy [11], [12].

### Identification of differentially expressed transcript units

Operons, as well as single genes that were not assigned to any operons, were regarded as transcript units (TUs). The average expression value of genes located in one same operon was adopted to characterize the expression level of the operon. EDGE and Timecourse, the most popular tools to analyze time-course microarray data, were applied to identify differentially expressed TUs [20]–[22]. Both methods rank TUs according to the significance of differential expression across time course.

### Classification of coexpressed TUs into modules

Coexpressed TUs in *S. erythraea* were detected by constructing weighted gene coexpression networks [23], [24]. First, a matrix of correlations between all differentially expressed TU pairs was built, and then transformed into an adjacency matrix using a power function where the connection strength between two TUs 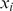 and 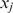 was formulized as 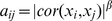. The parameter 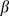 was determined such that the resulting adjacency matrix was approximately scale-free based on a model-fitting index [24]. This index was defined as the coefficient of determination (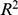) of the linear model constructed by regressing 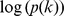 onto 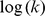, with 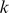 representing the degree of a given node and 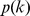 indicating the frequency distribution of the degree 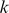 in the coexpression network. The model-fitting index of a perfect scale-free network was 1. We chose the smallest value of 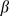 (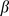 = 11) to make the model-fitting index 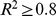 (Figure S2) [13], [24].

The adjacency matrix was further transformed into a topological overlap matrix to more readily identify modules of highly coexpressed TUs. The topological overlap captured not only the direct interaction between two TUs i and j but also their indirect interactions through all the other TUs in the network. Thus, A similarity measure was defined: 
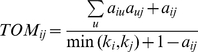
, where 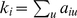 was the node connectivity [24], [25]. Subsequently, 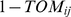 was used as a distance matrix in the hierarchical clustering of the transcript units for module detection [25].

### Classification of coexpressed modules into groups

The module eigengene, the first principal component of the matrix of expression values of a given module, was adopted to characterize the gene expression profile of the module [23]. The modules were then clustered according to their eigengenes with complete linkage method.

### Detection of associations among modules

Similar to the previous procedure when clustering modules into groups, module eigengene was still used to characterize the gene expression profile of a module.

The association between module pairs were estimated with partial correlation coefficient in order to reveal ‘direct’ correlations between two variables after removing the effects of other variables, and a network was then constructed based on Gaussian graphical model [26]–[28]. Summarily, supposing a linear relationship among variables can be described by a multivariate normal distribution, the partial correlation matrix provided dependence relationships among variables since a nonzero partial correlation between two variables indicated conditional dependence given all other variables; and a zero partial correlation indicated that the variables were conditionally independent. To be exact, given 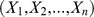, the partial correlation between 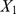 and 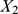 was defined as the correlation of 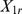 and 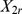 where 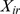 denoted the residuals obtained after regressing 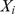 upon 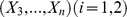.

## Supporting Information

Figure S1 Detected differential expressed TUs overlap between EDGE and Timecourse.
(0.09 MB PDF)

Figure S2 Analysis of network topology for various soft-thresholding powers. The left panel shows the scale-free fit index (y-axis) as a function of the soft-thresholding power (x-axis). The right panel displays the mean connectivity (degree, y-axis) as a function of the soft-thresholding power (x-axis).
(0.06 MB PDF)

Figure S3 Clustering of modules into groups.
(0.07 MB PDF)

Table S1 Analysis of module-trait (erythromycin production) associations.
(0.03 MB XLS)

Table S2 Comparison with the methods of Madeira (2009) and Kiddle (2010).
(0.05 MB XLS)
