# Supplementary figures and images for: Identifying Modules of Coexpressed Transcript Units and Their Organization of Saccharopolyspora erythraea from Time Series Gene Expression Profiles
Source: PLoS One. 2010 Aug 12;5(8):e12126. doi: 10.1371/journal.pone.0012126 (PMC2920828; doi:10.1371/journal.pone.0012126)

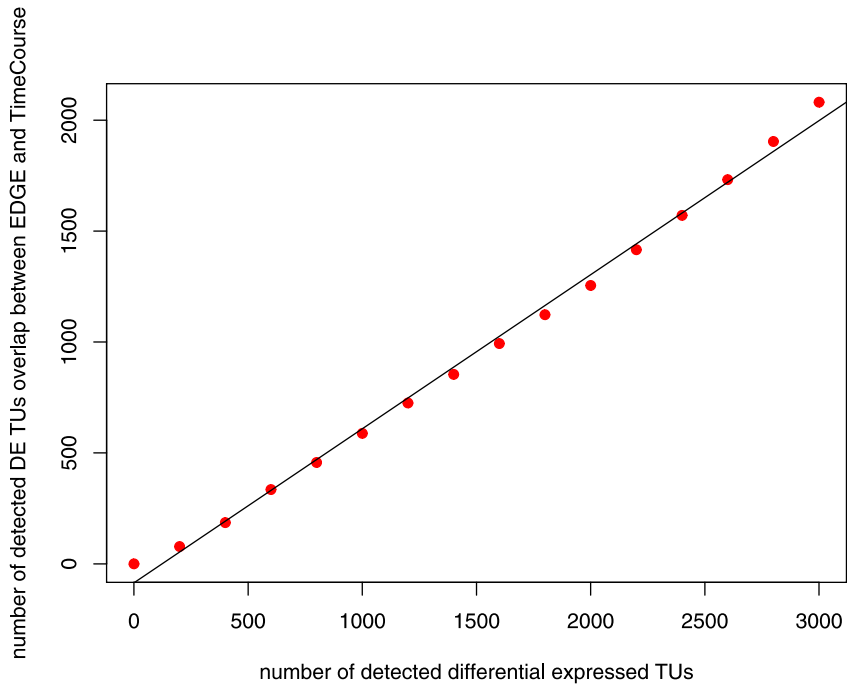

Supplement: Figure S1 — Detected differential expressed TUs overlap between EDGE and Timecourse. (0.09 MB PDF) [file pone.0012126.s001.pdf]

### Scale independence

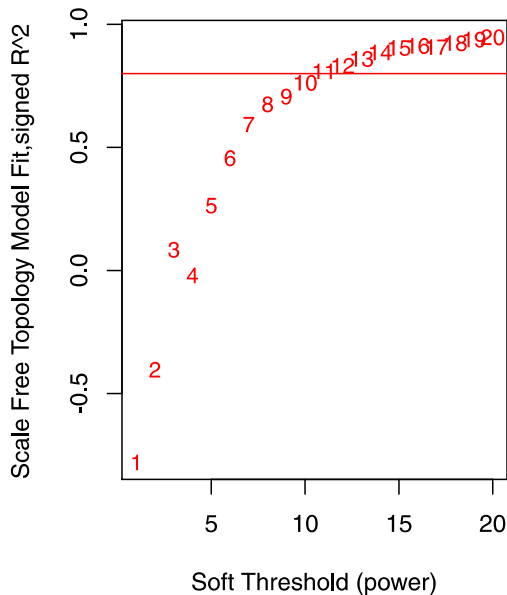

### Mean connectivity

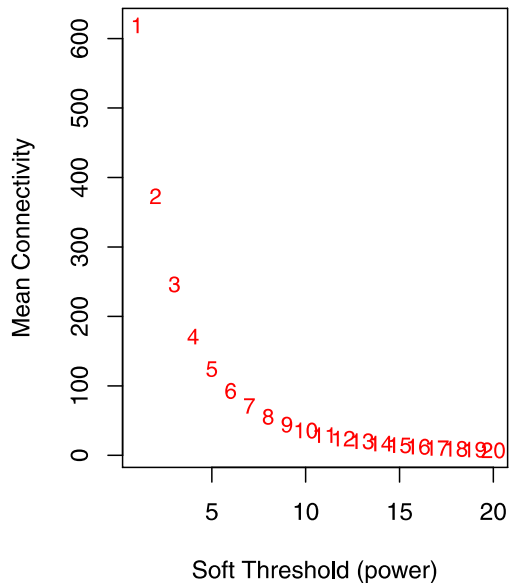

Supplement: Figure S2 — Analysis of network topology for various soft-thresholding powers. The left panel shows the scale-free fit index (y-axis) as a function of the soft-thresholding power (x-axis). The right panel displays the mean connectivity (degree, y-axis) as a function of the soft-thresholding power (x-axis). (0.06 MB PDF) [file pone.0012126.s002.pdf]

# Clustering of module eigengenes

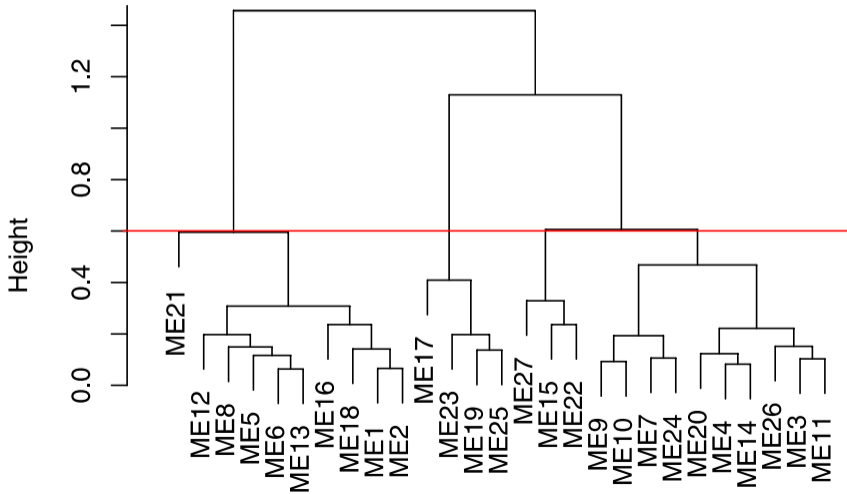

Supplement: Figure S3 — Clustering of modules into groups. (0.07 MB PDF) [file pone.0012126.s003.pdf]
